# Look past the cooperative eye hypothesis: reconsidering the evolution of human eye appearance

**DOI:** 10.1111/brv.70033

**Published:** 2025-05-14

**Authors:** Juan Olvido Perea‐García, Aurora Teuben, Kai R. Caspar

**Affiliations:** ^1^ Center for Language Evolution Studies Nicolaus Copernicus University in Toruń Fosa Staromiejska 3 Toruń 87‐100 Poland; ^2^ University Institute for Health and Biomedical Research (IUIBS), Universidad Las Palmas de Gran Canaria Las Palmas Spain; ^3^ University of Amsterdam Science Park 904 Amsterdam 1098 XH The Netherlands; ^4^ Institute of Cell Biology Heinrich Heine University Düsseldorf Universitätsstr. 1 Düsseldorf D‐40225 Germany

**Keywords:** pigmentation, photoprotection, primates, communication, intraspecific variation, sclera, conjunctiva

## Abstract

The external appearance of the human eye has been prominently linked to the evolution of complex sociocognitive functions in our species. The cooperative eye hypothesis (CEH) proposes that human eyeballs, with their weakly expressed conjunctival and scleral pigmentation, are uniquely conspicuous and evolved under selective pressures to behave cooperatively, therefore signalling attentiveness to conspecifics. Non‐human primates are instead assumed to display less‐salient eye morphologies that help mask their gaze to facilitate competitive, rather than cooperative actions. Here, we argue that the CEH, although continuing to be influential, lacks robust empirical support. Over the past two decades, multidisciplinary research has undermined its original rationale and central premises: human eye pigmentation does not uniquely stand out among primates, it is not uniform at species level and the available evidence does not conclusively suggest that it facilitates gaze following to notable extents. Hence, the CEH currently provides a theoretical framework that risks confusing, rather than informing, inferences about the evolution of human external eye appearance and its selective drivers. In a call to move past it, we review alternative hypotheses with the potential to elucidate the emergence of the human ocular phenotype from the considerable spectrum of diversity found within the primate order.

## I. INTRODUCTION

The external appearance of the human eye (i.e. the portion of the eyeball visible within the palpebral fissure) has been prominently stated to be unique among primates (Kobayashi & Kohshima 1997, 2001; Emery, 2000), owing to the conspicuous contrast between its iris and the widely exposed white of the eye that surrounds it. The latter is created by two different tissues, the sclera and the overlying bulbar conjunctiva that adheres to it. Although we will use the term “depigmented” in reference to the appearance of these peri‐iridal tissues in our species, we stress that humans still invariably harbour significant amounts of the pigment melanin, produced by melanocytes, in both their bulbar conjunctiva and sclera (Durairaj, Chastain & Kompella, 2012; Jakobiec, 2016). However, these peri‐iridal pigments are often not macroscopically visible. Although much of the relevant literature refers almost exclusively to the “colour of the sclera” (e.g. Tomasello *et al*., 2007; Mayhew & Gómez, 2015; Caspar *et al*., 2021; Mearing *et al*., 2022; Clark *et al*., 2023), it is actually the conjunctival epithelium which, if pigmented, most importantly determines the appearance of the peri‐iridal portion of the eyeball in humans and other mammals (Montiani‐Ferreira, Moore & Ben‐Shlomo, 2022). Peri‐iridal pigmentation or scleral appearance (i.e. determined by both the sclera and the bulbar conjunctiva) are thus more accurate terms that we will use throughout this article.

The eye morphology of our species has been proposed to be functionally interwoven with an array of uniquely human behaviours such as triadic joint action, ostensive communication, and language acquisition (e.g. Kobayashi & Kohshima, 2001; Tomasello *et al*., 2007). Accordingly, various hypotheses have attempted to explain its emergence. Of these, the most influential is the cooperative eye hypothesis (CEH; Tomasello *et al*., 2007). It proposes that selection pressures specific to the human lineage drove a loss of peri‐iridal pigmentation to enhance eye‐mediated social signalling. Thus, the CEH proposes that a unique propensity to cooperate led to a reduction of pigment in the peri‐iridal tissues, facilitating the perception of eye gaze direction by conspecifics (i.e. the reorientation of one's eyeballs, irrespective of head orientation, for example when glancing).

The CEH was primarily conceived on the basis of comparative morphological data provided by Kobayashi & Kohshima (1997, 2001), although a very similar idea was proposed earlier by Morris (1985), based on anecdotal observations. Kobayashi & Kohshima (1997, 2001) qualitatively examined video stills, photographs and eyeball specimens of 88 primate species to score the degree of peri‐iridal pigmentation and relative contrast between it and adjacent tissues, namely facial skin and iris. This resulted in a classification of primate eyes into four types, three of which were considered cryptic. Just the fourth type, which was characterized by depigmented peri‐iridal tissues, was considered conspicuous. Humans were the only species assigned to this type. Kobayashi & Kohshima (1997, 2001) argued that several purportedly unique features of the human eye (e.g. peri‐iridal depigmentation and high width‐to‐height ratio) made it an exceptionally effective organ for conveying eye gaze signals in social contexts (i. e. gaze signalling hypothesis). This idea was included and developed further in an influential review by Emery (2000) and later adopted by Tomasello *et al*. (2007) to explain observed differences in gaze‐following behaviour between captive non‐human great apes (from here on "great apes", if not specified otherwise) and human infants when presented with a human demonstrator. While gaze following in children as well as apes was influenced by both head and eye movement, the latter was found to be of notably greater importance for the human children. With reference to Kobayashi & Kohshima (1997, 2001) and similar to Emery (2000), Tomasello *et al*. (2007) argued that the striking appearance of the human eye evolved to enable referential communication based on subtle eye gaze signals alone and to facilitate joint attention, coining the name “cooperative eye hypothesis” (CEH) for this idea. The CEH singled out peri‐iridal depigmentation as an anatomical correlate of cooperativeness and, conversely, the presence of notable peri‐iridal pigmentation as a correlate of competitive behaviour. The latter was deemed typical for non‐human primates, which were hypothesized to benefit from concealing their eye gaze from conspecifics to avoid being exploited by them (Tomasello *et al*., 2007).

It is hard to measure the reach of the CEH accurately, but there is no doubt that it has been exceptionally influential within its rather short time of existence: it has deeply impacted a wide range of fields, motivating proposals within linguistics (e.g. Perniss & Vigliocco, 2014; Wacewicz *et al*., 2022), comparative, developmental, and clinical psychology (e.g. Herrmann *et al*., 2007; Senju, Csibra & Johnson, 2008; Segal, Goetz & Maldonado, 2016), palaeoanthropology and zoology (Hare, 2017; Caspar *et al*., 2021), philosophy (Kee, 2024) and human–computer interaction (Khoramshahi *et al*., 2016). In consequence, human ocular appearance is now often regarded as “a well‐established and widely accepted example of how the human body's appearance has evolved to facilitate cooperative sociality” (Kee, 2024).

**Fig. 1:**
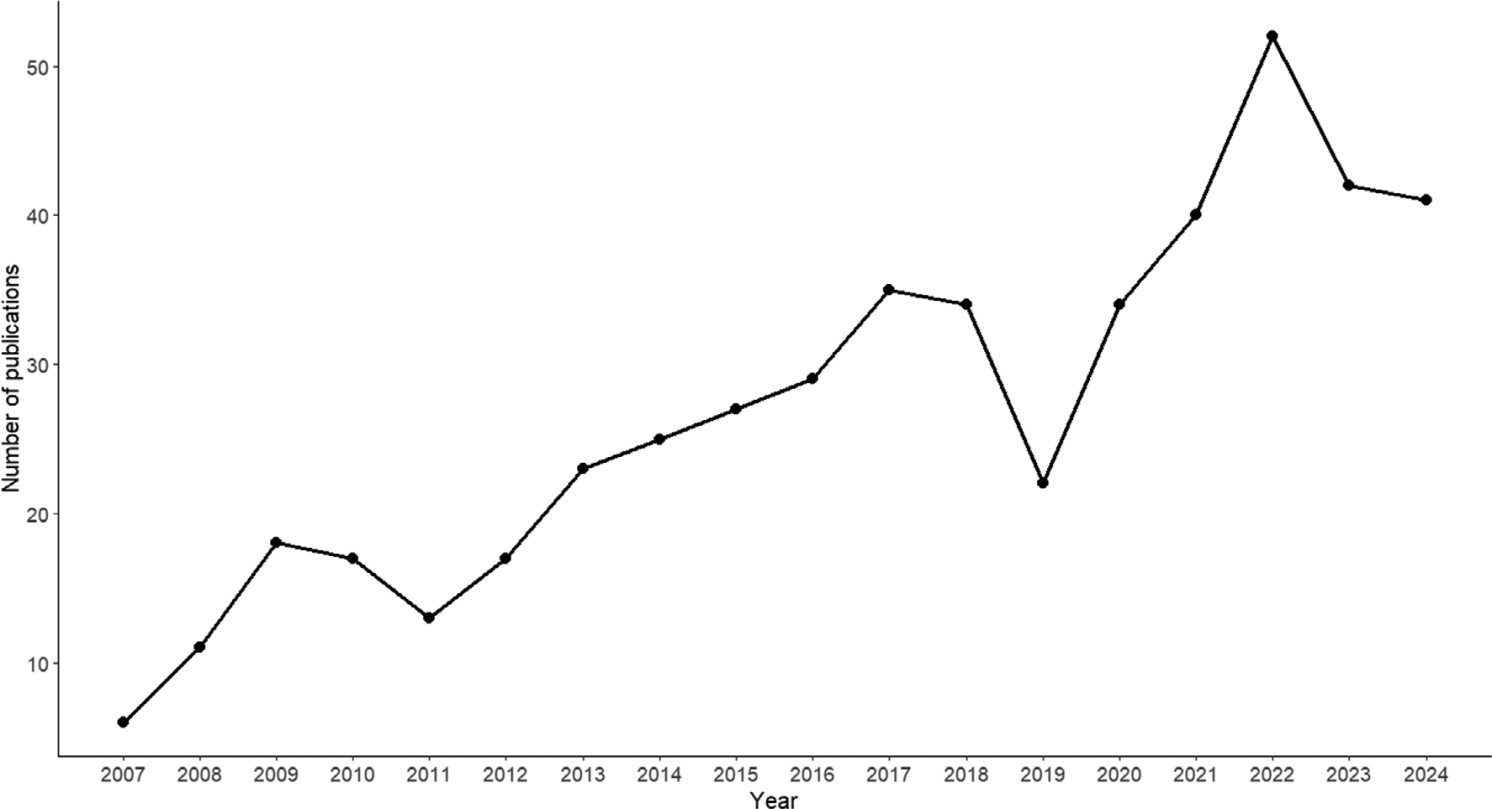
Occurrence of the expression “cooperative eye hypothesis” in academic publications published in the respective year according to *Google Scholar*. Data inspected on April 21st, 2025, results included only up to 2024. Data collected using code made available by Strobel (2018).

According to *Google Scholar* (May 2025), the relevant papers by Kobayashi & Kohshima (1997, 2001), Emery (2000) and Tomasello *et al*. (2007) have been collectively cited 4786 times (note that this is not the same as the number of individual publications citing these papers). Perhaps most importantly, however, the CEH has had a vast impact on popular science literature (e.g. Tomasello, 2018; Bregman, 2020; Hare & Woods, 2021; Hrdy, 2024), transcending academia and echoing through both traditional and digital media, including videos on platforms such as YouTube dedicated to the topic (e.g. EDGE Science, 2022), and its own Wikipedia article (“Cooperative eye hypothesis”, 2024). The number of academic publications using the phrase “cooperative eye hypothesis” follows an increasing trend since the term was coined in 2007 (Fig. 1). Hence, the CEH has had, and continues to have, an important impact on both academic and non‐academic thinking.

Here, we propose to reconsider the status of the CEH as a keystone idea because its central premises have either been greatly undermined, lack convincing support, or were based on flawed assumptions. We acknowledge that there are a number of traits highly characteristic for human eyes that set them apart from those of other primates. For example, human eyes are distinguished by a laterally widened palpebral fissure [Kobayashi & Kohshima, 1997, 2001; which, however, do not lead to a uniquely wide exposition of the scleral portion of the eye during sideways gazing, as is sometimes claimed (Mayhew & Gómez, 2015; Caspar *et al*., 2021)] and emotional tearing (Provine, Krosnowski & Brocato, 2009). Of outstanding relevance to the CEH, however, is the pigmentation of the human eye which is the focus of the present review. The CEH and its immediate derivatives (e.g. Kano, 2023) rest on four main premises, all of which are linked with peri‐iridal pigmentation: (*i*) human scleral appearance is unique among primates; (*ii*) it is morphologically uniform at the species level; (*iii*) it notably enhances gaze following; and (*iv*) its coevolution with social cognition is robustly supported by experimental data. We argue that none of these points are sufficiently supported by available evidence. Based on this critical assessment, we contest the suggestion of the CEH that “highly visible eyes […] are unique to humans, at least among the great apes […] because humans engage in special forms of cooperative/mutualistic interactions” (Tomasello *et al*., 2007, p. 319). Lastly, we discuss alternative scenarios about how human eye appearance might have evolved and which can be more easily reconciled with currently available data.

## II. THE FOUR KEY PREMISES OF THE CEH ARE NOT SUFFICIENTLY SUPPORTED

### (1) Human ocular depigmentation is not unique

**Fig. 2:**
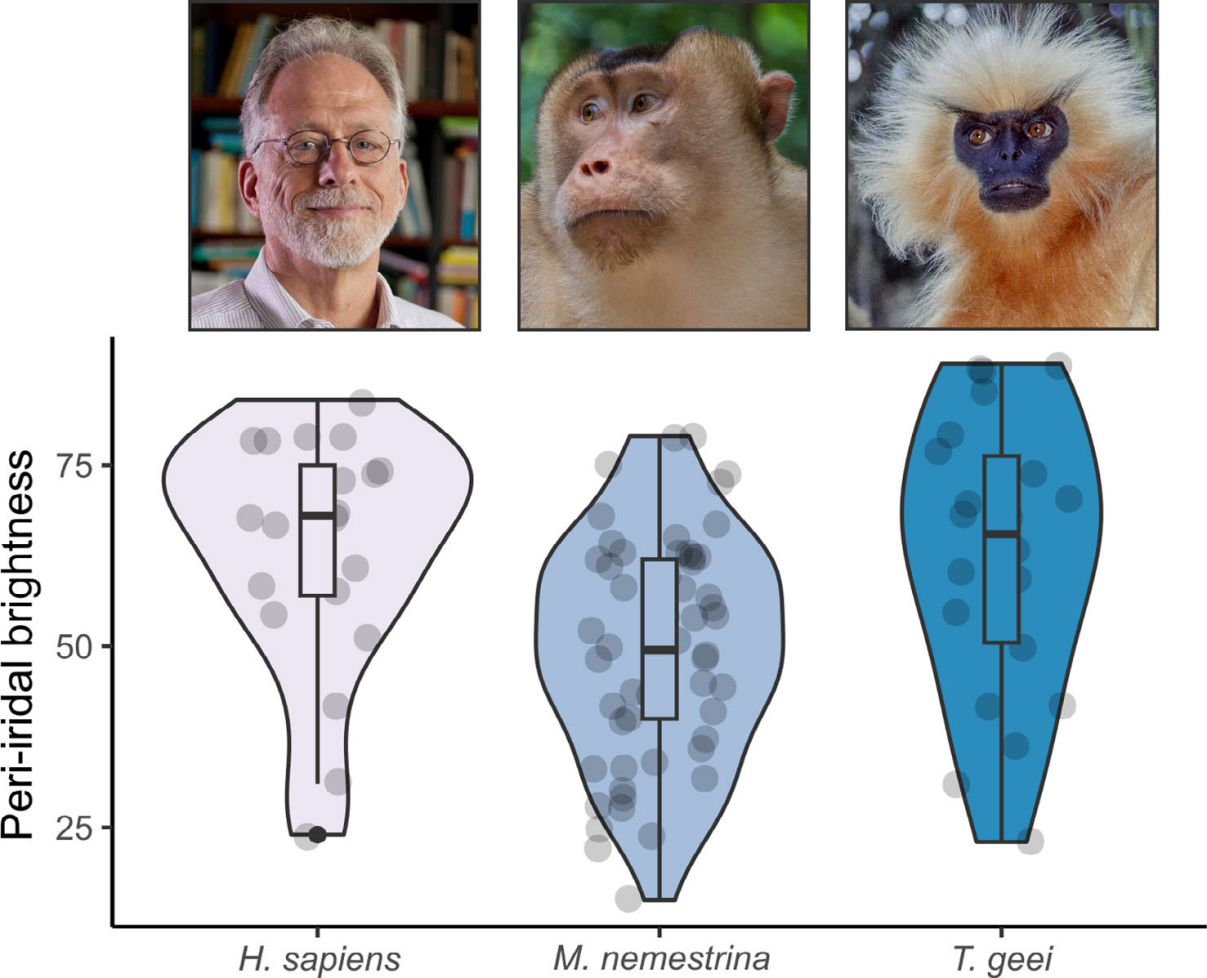
Brightness measurements of peri‐iridal tissues (sclera and overlying conjunctiva) taken from photographs of two non‐human primate species (golden langur *Trachypithecus geei*; southern pig‐tailed macaque *Macaca nemestrina*) that notably overlap with humans (*Homo sapiens*) in this regard [data for *T. geei* and *H. sapiens* from Perea‐García *et al*. (2022); data for *M. nemestrina* from Perea‐García *et al*. (2024); only adults were considered]. Photograph credits: *H. sapiens*: Duke University, used with permission; *M. nemestrina*: iNaturalist, Bridgette Gower; *T. geei*: iNaturalist, Ernst Hüttinger. Non‐human primate images are licensed under CC BY‐NC (https://creativecommons.org/licenses/by‐nc/4.0/).

**Fig. 3:**
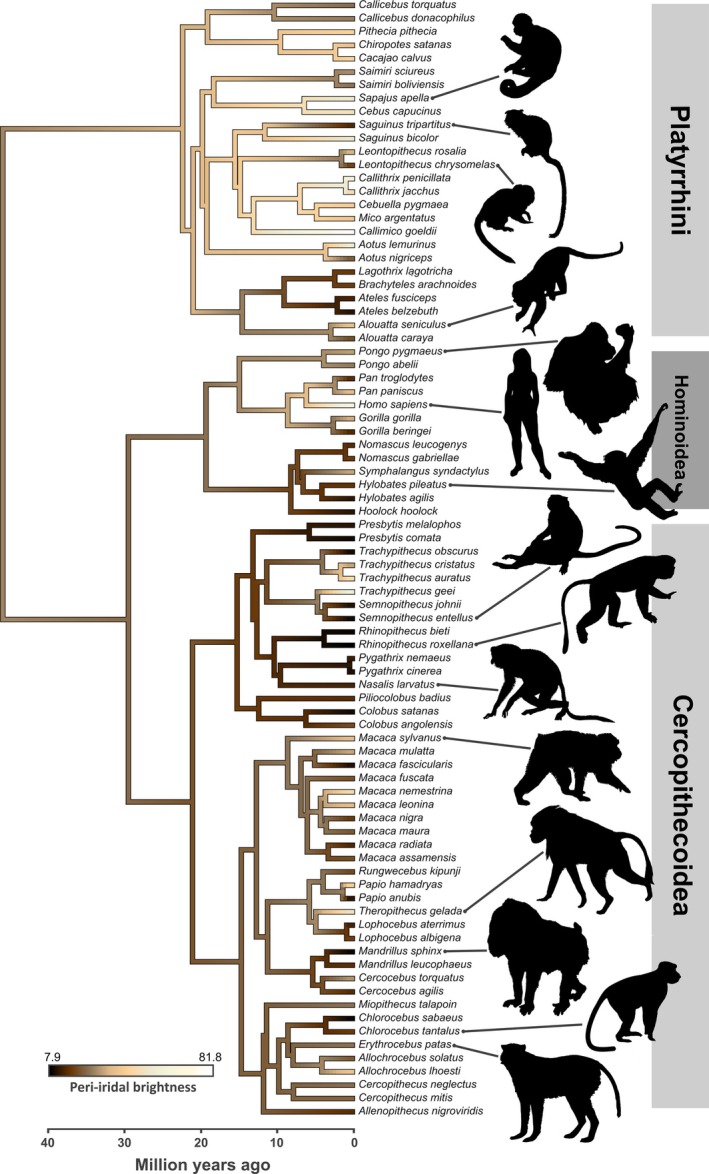
Reconstruction of ancestral peri‐iridal pigmentation in anthropoid primates based on species‐averaged brightness [hue‐saturation‐brightness (HSB) colour space] value measurements taken from digital photographs (maximum likelihood estimates assuming a Brownian motion model of evolutionary change). Colour is approximated. Note that weak pigmentation in humans was acquired secondarily but that several lineages of non‐human primates evolved similarly depigmented eyes convergently. Human data incorporated in this graphic primarily derive from white Eurasians and must therefore be interpreted with caution. Data from Perea‐García *et al*. (2022, 2024). Silhouettes derived from Phylopic. Credits: Kai R. Caspar – *Hylobates*, *Leontopithecus*, *Macaca*, *Saguinus*, *Sapajus*, *Semnopithecus*, *Theropithecus*; remaining silhouettes are in public domain. Figure created using the *contMap()* function in the R package *phytools* (Revell, 2012).

The CEH relies on the assumption that macroscopically depigmented peri‐iridal tissues are uniquely human. However, recent studies have accumulated abundant evidence to the contrary. Primates display a wide but gradual spectrum of peri‐iridal pigmentation patterns and humans do not monolithically stand out among this diversity (Figs 2 and 3). Comparative studies on external eye appearance have produced ample data demonstrating that numerous non‐human primates can display total or partial macroscopic depigmentation of peri‐iridal tissues, and thus show notable phenotypic overlap with humans either at the individual or even the species level [Mayhew & Gómez, 2015; Perea‐García, 2016; Perea‐García *et al*., 2019; Caspar *et al*., 2021; Clark *et al*., 2023; e.g. compare human peri‐iridal brightness with that of the golden langur *Trachypithecus geei* (Perea‐García *et al*., 2022) and southern pig‐tailed macaque *Macaca nemestrina* (Perea‐García *et al*., 2024) in Fig. 2]. Whereas well‐visible conjunctival pigmentation was probably present in the common ancestor of anthropoids, a bright scleral appearance evolved convergently multiple times among different primate lineages (Perea‐García *et al*., 2022; Fig. 3), including some populations of chimpanzees (*Pan troglodytes*; Clark *et al*., 2023), a species that is widely considered competitive rather than cooperative (Burkart, Hrdy & Van Schaik, 2009). Intraspecific variation in both human (see Section II.2) and non‐human primate eyeball conspicuity remains underreported [but see Mayhew & Gómez (2015) and Clark *et al*. (2023)] which might further obscure overlaps in peri‐iridal phenotypes between them. Kobayashi & Kohshima's (1997, 2001) clear‐cut categorical classification into cryptic and conspicuous morphologies in conjunction with the small intraspecific sample sizes underlying their work has likely contributed to the erroneous assumption of uniform eye appearance in a given species. The CEH cannot make sense of the diverse ocular phenotypes we find among the primate order (Caspar *et al*., 2021).

However, while acknowledging the aforementioned spectrum of ocular phenotypes, some recent works still argue that humans are exceptional among primates for displaying a uniformly white scleral appearance without notable inter‐individual variation (Kano *et al*., 2022a; Kano, Kawaguchi & Hanling, 2022b; Kano, 2023). This assumption must be reconsidered because first, intraspecific variation in human ocular phenotypes seems to be greater than is commonly assumed in the socio‐cognition literature (see Section II.2) and second, several other non‐human primates appear to display striking uniformity of bright‐eyed phenotypes (Fig. 2; Mearing *et al*., 2022; Perea‐García *et al*., 2022, 2024). For instance, Oriá *et al*. (2013) reported that several species of platyrrhine monkeys (*Callithrix jacchus*, *N* = 31; *Callithrix penicillata*, *N* = 8; *Cebus*/*Sapajus* spp., *N* = 22; *Sapajus xanthosternos*, *N* = 9) did not display any macroscopic pigmentation in their conjunctivae, even upon close examination.

### (2) Human ocular depigmentation is not uniform

Because current data no longer support the notion of human uniqueness based on peri‐iridal depigmentation alone, current proponents of the CEH argue that it is the degree and uniformity of such depigmentation that sets us apart from other primates (Kano *et al*., 2022a, 2022b; Kano, 2023; see also Hare & Woods, 2021). As pointed out above, this assumption does not consider the observation that ocular phenotypes in various bright‐eyed monkeys can be strikingly homogeneous (Oriá *et al*., 2013; Perea‐García *et al*., 2022, 2024) and so far, its proponents have not established what constitutes “uniformity” at the species level. It is also worth pointing out that the quantitative evidence on global variation in human peri‐iridal pigmentation necessary to make such a claim in a compelling fashion is currently unavailable.

**Fig. 4:**
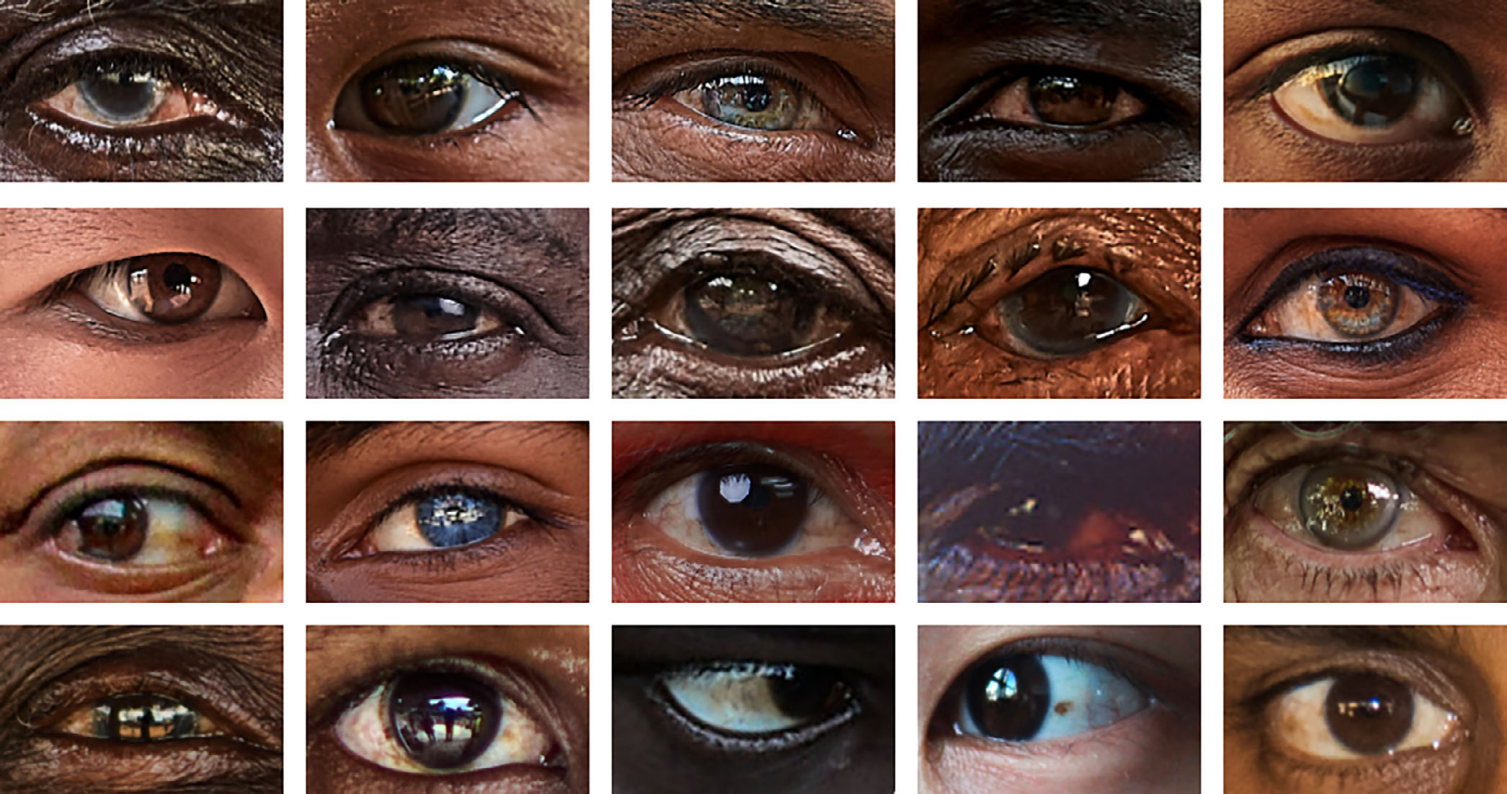
An impression of the diversity of peri‐iridal pigmentation found among modern humans. All photographs by Marios Forsos unless indicated otherwise. Note that the ethnic background of the persons concerned was often not known to us so that we only indicate the state in which the photograph was taken. Left to right, top to bottom ‐ 1. row: Kenya, Myanmar, Bangladesh, Ethiopia, Mali, 2nd row: Mongolia, Tanzania, Mali, Vietnam, India, 3rd row: India (photograph by Surabhi Vijayaraghavan, CC BY‐SA 4.0, https://creativecommons.org/licenses/by‐sa/4.0/deed.de), India, Ecuador, Australia (photograph by Gunther Deichmann, used with permission), Ladakh (India/Pakistan), 4th row: Ivory Coast, Ethiopia, Singapore (Chinese ancestry; photograph by JOPG), India (photograph by Rita Willaert, CC BY‐NC‐SA 4.0, https://creativecommons.org/licenses/by‐sa/4.0/deed.de). [Correction added on 20 May 2025, after first online publication: In the caption of Figure 4, the description of the 4th row has been corrected.]

The popular notion that, at the species level, human peri‐iridal tissues are completely and homogeneously depigmented (Hare & Woods, 2021; Kano, 2023) might partly derive from a critical bias – both the researchers and the human subjects featured in available studies are almost exclusively urbanites of Northern Eurasian descent. However, there is substantial non‐pathological variation in conjunctival pigmentation across the human species, especially when considering rural and indigenous populations living near the equator in Africa and Asia, as well as native Australian ethnicities (Fig. 4, see Section III.1 for why stronger pigmentation in these groups is expected).

Notable variation in peri‐iridal pigmentation among human populations, while still underexplored, is undeniable. Interestingly, said variation was reported in the literature decades before the emergence of the CEH (Mann, 1966). A general positive covariation of epidermal and conjunctival pigmentation in humans is well documented in the medical literature (e.g. Mann, 1966; Jakobiec, 1984, 2016; Singh *et al*., 1998; Blake, Lai & Edward, 2003; Whittington *et al*., 2024). It appears intuitive that ocular and epidermal pigmentation are correlated within individuals, but quantitative studies on the linkage between these traits are still lacking. Interestingly, the pigment‐producing melanocytes of the sclera and uvea derive from different embryonic precursors than those of the skin and are physiologically distinct from them (Boissy & Hornyak, 2006) but the ontogenetic origins of conjunctival pigment cells remain unclear (discussed by Caspar, Hüttner & Begall, 2023). Hence, the regulation of pigment production in epidermal and conjunctival melanocytes could differ in important respects.

Macroscopically visible pigmentation of the conjunctiva in humans is primarily caused by elevated pigment production in conjunctival melanocytes compared to phenotypes with transparent conjunctivae, rather than by an increase in melanocyte number (Jakobiec, 1984, 2016). The resulting brownish, often patchy coloration is almost always bilateral (although not necessarily symmetric) and is known as intraepithelial non‐proliferative melanocytic pigmentation or complexion‐associated melanosis (traditionally termed “benign epithelial melanosis” or “racial melanosis”). Available data suggest a widespread macroscopic manifestation of this condition in darker‐skinned ethnicities, especially older adults, with an incidence of over 90% reported for African Americans (Singh *et al*., 1998) and corresponding qualitative descriptions for native Australians (Mann, 1966). It does not represent a pathological phenomenon, nor is it linked to an increased risk of conjunctival melanoma (e.g. Oellers & Karp, 2012). Instead, it simply reflects part of the notable spectrum of scleral appearance in our species. The latter also covers the occurrence of naevi in the episclera (benign freckle‐like growths; Jakobiec, 2016), pigmented scleral emissaries (nerve or blood vessel‐associated accumulations of pigment; Blake *et al*., 2003) and age‐related staining of the peri‐iridal tissues (Russell *et al*., 2014). Nevertheless, despite the substantial body of available research, there is still a crucial lack of comparative quantitative data on intraspecific patterns in human eye pigmentation. This issue urgently needs to be addressed to set human and non‐human primate variation properly into perspective.

In short, humans display heterogenous, not uniform depigmentation of peri‐iridal tissues at species level, thus undermining recent attempts to “rescue” the categorical uniqueness of the human eye (Kano, 2023) that served as a rationale to propose the CEH in the first place (Tomasello *et al*., 2007).

### (3) The human ocular phenotype does not notably enhance gaze following

The CEH assumes that depigmentation of the human eyeball is crucial for facilitating effective gaze following by conspecifics. However, accumulating evidence shows that ocular appearance in great apes allows for effortless eye‐gaze following as well [except under very challenging lighting conditions (*cf*. Kano *et al*., 2022a; Whitham *et al*., 2022a)], critically undermining the idea that these primates exhibit “gaze camouflage” (Kobayashi & Kohshima, 1997, 2001; Tomasello *et al*., 2007). For instance, models for chimpanzee visual perception suggest that their eye gaze direction can be conspicuous to conspecifics over distances of more than 20 m (Whitham *et al*., 2022a). Indeed, human observers can infer chimpanzee eye gaze direction over distances of at least 10 m when watching live animals (Bethell, Vick & Bard, 2007; note that this study did not attempt to code gaze direction over longer distances). Supposedly cryptic non‐human primate eyes can thus effectively convey eye gaze signals over considerable ranges.

Experimental work showing humans photographs of conspecifics with or without artificially darkened peri‐iridal tissues suggests only a minor signalling advantage of the bright‐eyed phenotype. Although it takes humans significantly longer to judge the eye gaze direction of darkened compared to naturally coloured eyes, the time differences only encompass fractions of a second and there are no notable differences in the accuracy of deducing eye gaze direction from normal human models and those with matched iris and peri‐iridal colours (Yorzinski & Miller, 2020). It should also be noted that the tested participants were naïve to the dark‐eyes stimulus but acquainted with typical human eyes, so that this difference might simply relate to their familiarity with human‐typical eye appearance and not sensory limitations or innate cognitive biases. The aforementioned lack of familiarity can also parsimoniously explain the recent findings of Wolf, Thielhelm & Tomasello (2023), which were interpreted as supporting the CEH. This study reported that human children show cooperative preferences for fictional humanoid characters with bright eyes over otherwise exact copies with dark peri‐iridal complexion.

Some studies that continue to favour the CEH acknowledge the limited evidence for superior salience and point out that human eyes may only provide specific advantages for eye gaze signalling over considerable distances (the original CEH framework focused on close‐range dyadic interaction) and under challenging visual conditions (Yorzinski & Miller, 2020; Kano, 2023). For instance, it has been speculated that cooperative hunting could benefit from such signals (Yorzinski & Miller, 2020), but we are unaware of data that would suggest that eye gaze, rather than head and body orientation and movement, critically aids in coordinating actions during group hunting in humans. In this context, it is worth considering that work using pictorial cues in laboratory settings indicates that human eye gaze direction can only be reliably interpreted over a range of 10–20 m (Watt, Craven & Quinn, 2007); distances for which chimpanzee eyes might be suitable signalling devices as well (Whitham *et al*., 2022a). However, these findings require replication under naturalistic conditions, including different lighting regimes. Therefore, while data suggestive of a measurable signalling advantage of bright peri‐iridal tissues under challenging visual conditions are available (Yorzinski & Miller, 2020; Kano et al., 2022a, Kano *et al*., 2022b), we believe more evidence is needed before we can conclude that this has a significant impact on real‐life scenarios.

Irrespective of pigmentation, eye gaze in humans and non‐humans may be effectively communicated by more subtle and understudied phenomena such as changes in the palpebral fissure in response to eye movements, the angle of the pupil (Prein *et al*., 2024) or light reflections on the cornea (Anstis, 2018). The latter is often overlooked in studies using artificially altered images of eyes, including those arguing in favour of the CEH [Kano *et al*. (2022b) inverted the colour of the corneal reflex and the pupil; Yorzinski & Miller (2020) removed the corneal reflex from test images]. However, this could have biased these studies' outcomes as other experiments suggest a relevance of such reflections when it comes to interpreting eye gaze [Antsis (2018) – but note that work with more naturalistic stimuli is required to bolster these findings]. Finally, most studies so far have relied on static stimuli, but humans can recruit eye movements in addition to static cues at least to distinguish between competing referents (Anderson, Risko & Kingstone, 2016).

The mere observation that human eyes are salient in dim light should not convince us that they evolved to provide directional cues under such challenging conditions (Kano, 2023) because eye gaze conspicuousness alone is not a reliable indicator for whether a species routinely perceives eye gaze cues as referential signals. Capuchins (*Cebus*/*Sapajus* spp.) for instance have very bright peri‐iridal tissues (Oriá *et al*., 2013; Perea‐García *et al*., 2022), and a recent study simulating the vision of *Sapajus apella* strongly suggests that their ocular morphology is conspicuous to conspecifics (Whitham *et al*., 2022b). Still, there is no evidence so far indicating that they are sensitive to eye gaze direction as a referential cue or that they can even learn to interpret it as such from a human demonstrator (Vick & Anderson, 2000). The eyes of chimpanzees are also notably conspicuous to conspecifics (Kano *et al*., 2022a; Whitham *et al*., 2022a), yet these apes struggle to extract information about eye‐gaze direction from photographs. Recently, Kano *et al*. (2022b) reported that even extensively trained laboratory‐housed chimpanzees have great problems responding to pictorial eye gaze cues of both humans and conspecifics. The authors attempted to train chimpanzees to distinguish between face stimuli displaying either direct or averted eye gaze. Although some chimpanzees received more than 100 training sessions, only three of the 10 original subjects eventually succeeded in the task and were more proficient at distinguishing eye gaze direction in pictures with bright peri‐iridal tissues (Kano *et al*., 2022b; but note that, very importantly, the cues presented were not embedded in a relevant social context). In line with that, chimpanzees view facial pictures of conspecifics differently from humans (residing in Japan and Germany) in that they pay significantly less attention to the eyes and more to, for instance, the mouth (Kano & Tomonaga, 2010). Eye‐tracking studies in Western gorillas (*Gorilla gorilla*) and Sumatran orangutans (*Pongo abelii*), which often display bright peri‐iridal tissues more akin to humans (Mayhew & Gómez, 2015; Perea‐García, 2016; Caspar *et al*., 2021), revealed viewing patterns similar to that of chimpanzees (Kano, Call & Tomonaga, 2012). While more studies need to be conducted before solid conclusions can be drawn, the connection between ocular conspicuity and attention to eye‐mediated stimuli is not as straightforward as is typically assumed. To conclude, we lack compelling evidence that human eye appearance does notably enhance eye‐gaze signalling compared to phenotypes more typical for great apes. Furthermore, while the literature since Kobayashi & Kohshima (1997) has mostly assumed that a species' ocular conspicuity equals reliance on ocular cues for communicative purposes, this relationship is not evident – species with conspicuous eyeballs may not utilize them in a fashion analogous to humans.

### (4) The CEH is not robustly supported by experimental data

Given the phenomenal reception of the CEH, one should expect the experimental data presented in the study of Tomasello *et al*. (2007) to be robust. However, there are several methodological shortcomings that need to be considered when evaluating the study's results and impact. The key finding of the study is that tested African great ape subjects (chimpanzees, *N* = 11; bonobos, *Pan paniscus*, *N* = 4; Western gorillas, *N* = 4) housed at the Wolfgang Köhler Primate Research Center at Zoo Leipzig were more prone to following the head, rather than the eye gaze of human experimenters than a cohort of WEIRD (Western, Educated, Industrialized, Rich and Democratic; Henrich, Heine & Norenzayan, 2010) human infants. The difference in eye gaze‐following behaviour between human infants and great apes was interpreted to result from derived socio‐cognitive adaptations in humans compared to great apes. Yet, as is the case with many works focusing on human *versus* great ape cognition (Leavens, Bard & Hopkins, 2019), the study design does not allow us to rule out that these differences were instead prompted by the testing paradigm and the subjects' developmental histories. First, for practical reasons, the testing environments were different between apes and infants, with only the ape subjects being tested in isolation from conspecifics and separated from the experimenter by a barrier (a plexiglas pane or a wire mesh). While barriers are accepted as pragmatic limitations for working with captive apes, they might have biased their task performance, as experiments with domestic dogs (*Canis familiaris*) suggest [Kirchhofer *et al*., 2012; Clark & Leavens, 2019; but see Clark, Flack & Leavens (2020) for a lack of barrier effects on test performance in human children]. Second, whereas the human children responded to a conspecific providing gaze signals, the apes had to work with a heterospecific cue giver. Thus, the stimuli were not comparable and potentially inadequate for the apes. Indeed, a later study at the same location reported that chimpanzees (in contrast to bonobos and orangutans) did not follow the combined head and eye orientation of a human to a target at notable frequencies when viewing a video, but did so with a conspecific stimulus (Kano & Call, 2014). The same study also showed that, *vice versa*, human infants did not successfully follow the gaze of great apes. Interestingly, apes that receive adequate exposure to human social habits [something that was arguably not the case for the subjects tested by Tomasello *et al*. (2007) which were well‐acquainted with experimental set‐ups but still non‐enculturated “zoo apes”], can reliably interpret human eye gaze as a referential cue (Itakura & Tanaka, 1998; Inoue, Inoue & Itakura, 2004; Caspar *et al*., 2018) and thus might react differently in gaze‐following assays. An ape's prior experience will have strong effects on its performance in such cognitive tasks (Leavens, Bard & Hopkins, 2010; Leavens *et al*., 2019). We are unaware of studies that tried to replicate the findings of Tomasello *et al*. (2007) in a similar paradigm or one that addresses its aforementioned shortcomings. Whereas this might appear surprising given the work's impact, a very low replication rate is unfortunately typical for primate cognition research (ManyPrimates *et al*., 2019). Given all these issues, we cannot consider the findings and inferences of Tomasello *et al*. (2007) compelling.

While there is evidence that non‐human anthropoid primates are sensitive to the eye gaze of conspecifics irrespective of head orientation (Deaner & Platt, 2003), few if any data on their ability to exploit eye gaze direction as a referential signal in typical social contexts are available (Caspar *et al*., 2021; but see Kaplan & Rogers, 2002). Yet, in experimental studies with human cue‐givers, diverse non‐human primates have at times been shown to follow eye gaze reliably independent of head orientation (e.g. Povinelli & Eddy, 1996; Ferrari *et al*., 2000; Burkart & Heschl, 2006; Tomasello *et al*., 2007). The scarcity of available information does not allow us to accept or dismiss a notable role of eye gaze in non‐human primate conspecific communication and substantial inter‐ and intraspecific variation must be expected (Kaplan & Rogers, 2002). At the same time, a nuanced perspective on our own species needs to be maintained: while eye gaze can doubtlessly convey highly relevant signals in human communication (e.g. Emery, 2000; Kano *et al*., 2012), we need to consider experimental evidence suggesting that humans still crucially rely on head directional cues when determining the gaze of others. This fact is not contested by the CEH but is frequently overlooked by secondary sources discussing it. Numerous experimental studies show that head orientation and other facial features remain important factors affecting perceived gaze in our species (e.g. Gamer & Hecht, 2007; Riccidardelli & Driver, 2008; Todorović, 2009) and are not simply overridden by eye orientation when they do not align with the latter directionally (Laube *et al*., 2011).

It is also worth noting that we still lack compelling evidence that relevance of eye gaze as referential cues is conserved across human populations. Recent cross‐cultural studies suggest that basic aspects of eye‐gaze following (Prein *et al*., 2024) and its development (Bohn *et al*., 2024) are indeed broadly shared across cultures, but like Tomasello *et al*. (2007), developmental psychologists have often made claims of universality from results obtained in WEIRD populations (Keller, 2018). Yet, early experiences, including cultural differences in face‐to‐face interactions, affect eye gaze‐following behaviours (Astor & Gredebäck, 2022). Even where categorical similarities have been found between cultures, gradual differences can still be observed, for instance in the accuracy with which infants follow eye gaze across development (Bohn *et al*., 2024).

Considering the overall scarcity of comparative data and the limitations of the study conducted by Tomasello *et al*. (2007), we still lack compelling information to determine how (un)important eye gaze is for great apes and other primates compared to humans. Further experimental evidence is urgently needed.

## III. DISCUSSION: ALTERNATIVE AVENUES FOR RESEARCH

In Section II, we critically examined the evidence used to support the CEH, arriving at the conclusion that none of its central premises has compelling support. Thus, we challenge the validity of the CEH and its status as a keystone idea in the field of comparative social cognition. Nevertheless, what it undoubtedly and importantly achieved was to generate broad interest in the evolution of ocular appearance in humans and other primates. The research it inspired gave rise to several alternative hypotheses on the evolution of primate ocular appearance that move beyond a species' propensity to cooperate or to compete. As is the case for pigmentation in general, multiple potential factors instead of one single cause should be considered to make sense of the spectrum of phenotypes that researchers have characterized since the work of Kobayashi & Kohshima (1997). Among these are photoregulation, sexual selection, and non‐referential communicative needs. How can hypotheses relating to these factors (which are not mutually exclusive) help to advance our understanding of the diversity of ocular pigmentation patterns in the primate order and in humans in particular?

### (1) Photoregulation

**Fig. 5:**
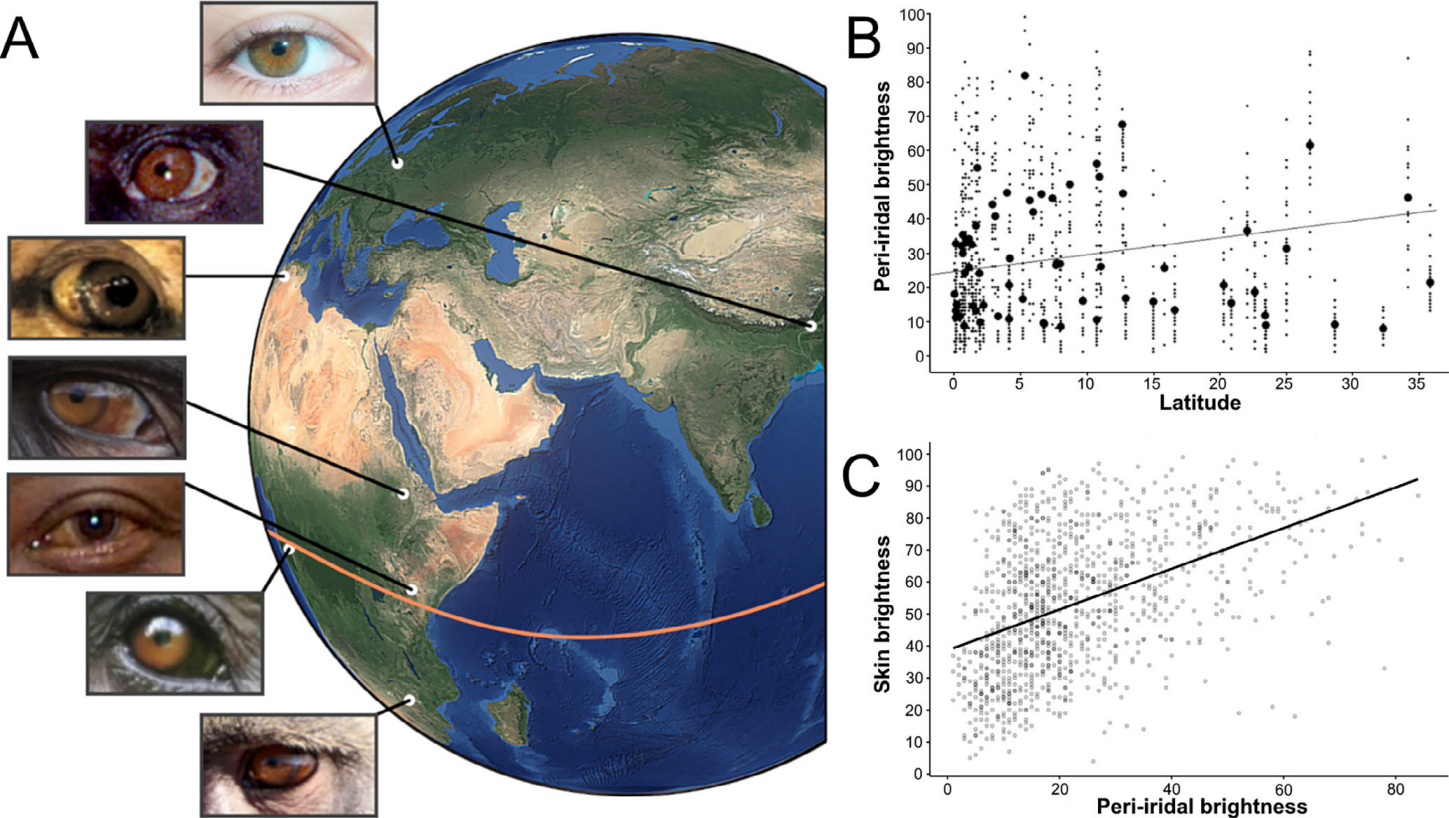
Peri‐iridal pigmentation in primates is indicative of photoregulatory needs. (A) Exemplar ocular phenotypes of catarrhine primates across latitudes, the equator is shown as an orange line. From top to bottom: *Homo sapiens* (Europe; public domain), *Trachypithecus geei* (by Ernst Hüttinger ‐ CC BY‐NC, https://creativecommons.org/licenses/by‐nc/4.0/), *Macaca sylvanus*, *Theropithecus gelada*, *Homo sapiens* (East Africa; public domain), *Pan troglodytes*, *Papio ursinus* (by flowcomm. CC BY 2.0, https://creativecommons.org/licenses/by/2.0/deed.en). Primate images taken from Perea‐García *et al*. (2022), if not otherwise indicated. Globe model by Pavel Matoušek (CC BY‐NC, https://creativecommons.org/licenses/by/4.0/). (B) Relationship between peri‐iridal pigmentation and latitude (mean of species range) in anthropoid primates. Taken from Perea‐García *et al*. (2022). (C) Relationship between peri‐iridal pigmentation and skin brightness in individual macaques (genus *Macaca*). Data from Perea‐García *et al*. (2024).

One idea that has recently gained traction is that primate eye pigmentation is shaped by photoregulatory needs (Fig. 5; Perea‐García *et al*., 2022). Melanin contributes to shielding the eye from harmful ultraviolet (UV) light and hence enables proper ocular functionality. Even small changes to the structure of the eye can alter the quantity and quality of light that penetrates the eyeball (Nischler *et al*., 2013; Renzi‐Hammond & Hammond, 2022; Bashkatov *et al*., 2010), and because vision is of exceptional importance in anthropoid primates (Kirk, 2004), it is unlikely that alterations become fixed if they disrupt visual function.

In the physiological and clinical literature, photoprotective adaptations of the eye have long been acknowledged (e.g. Kirschfeld, 1982). The bulbar conjunctiva and corneal limbus have to withstand significant UV exposure, which has been associated with the occurrence of detrimental conditions such as pterygia (MacKenzie *et al*., 1992). Both of these tissue types harbour sensitive stem cells critical to uphold tissue integrity and function (Ramos, Scott & Ahmad, 2015). UV exposure may be substantial for both the temporal and the nasal aspect of the eye: although the latter is shaded more effectively by the orbitae and nose bridge in many primates, it receives reflected light from the eye's temporal side which is focused onto the nasal portion of the eyeball by the cornea (Coroneo, Müller‐Stolzenburg & Ho, 1991; Notara *et al*., 2018). This asymmetrical and perhaps counterintuitive pattern of exposure to UV has been suggested to explain the greater abundance of pigment on the temporal side of primate eyeballs (Perea‐García, Danel & Monteiro, 2021; Perea‐García *et al*., 2024): a darkened temporal aspect of the eye absorbs light more effectively, so that the cornea will focus fewer photons onto the nasal aspect. That way, the risk of acquiring pathologies linked to high UV exposure, such as pterygia, might be ameliorated (e.g. Gazzard *et al*., 2002).

Available data suggest that the intensity of protective melanin pigmentation of peri‐iridal tissues has evolved in response to their exposure to UV light both within and among primate species: geographic latitude, which correlates negatively with UV irradiation, is a predictor of conjunctival pigmentation across monkeys and non‐human apes (Fig. 4B; Perea‐García *et al*., 2022). Species living closer to the equator tend to exhibit more pronounced peri‐iridal pigmentation, which aligns with trends previously reported for pigmentation patterns of the anthropoid face, especially the orbital region (Santana, Lynch Alfaro & Alfaro, 2012; Santana *et al*., 2013). In humans, the primate species with the widest geographical range, ophthalmological data also indicate that populations originating from low latitudes show stronger conjunctival pigmentation (e.g. Mann, 1966; Jakobiec, 1984; Singh *et al*., 1998) as well as other molecular adaptations to high UV irradiation (Kirschfeld, 1982). One contiguous effect of increased peri‐iridal pigmentation is protection from malignant tumours forming in those tissues (Hu, 2005). Indeed, the incidence of conjunctival cancers is negatively correlated with latitude in humans (Yu, Hu & McCormick, 2006) and notably lower in dark‐skinned ethnicities, which harbour more protective pigment in their ocular epithelia compared to lighter‐skinned ones (Culp *et al*., 2021). Apart from latitude alone, canopy cover, the use of specific forest strata (Dominy & Melin, 2020) and ground reflectivity (Perea‐García *et al*., 2024) may contribute substantially to UV exposure in primates, yet these factors have so far remained unexplored as drivers of ocular pigmentation. When considering proximate rather than ultimate drivers of ocular pigmentation patterns, it would be interesting to conduct comparisons among populations, or ideally between twins (see Sanfilippo *et al*., 2015), from different localities or habits related to prolonged UV exposure. It is currently unclear to what extent an individual's conjunctival pigmentation might change adaptively in response to UV irradiation over the course of life (conjunctival tanning). One study has compared the peri‐iridal pigmentation of office workers and farmers in Austria (Schmid‐Kubista *et al*., 2010). While no difference in pigmentation between these groups was reported, farmers showed a significantly greater incidence of tumours in the eyelid and conjunctiva. It could be that the Austrian participants in the study simply had no capacity to synthesize enough conjunctival melanin to be macroscopically detectable or that the coarse scoring system employed by the authors was not sensitive enough to identify potentially minute gradual changes in coloration. In conclusion, peri‐iridal pigmentation in various primate species can be explained as a photoregulatory adaptation to their lifestyles rather than resulting from a need for “gaze camouflage”.

However, a secondary reduction in pigmentation could suggest relaxation of such selective pressures. Regardless of latitudinal range, it is apparent that small‐bodied ‐ and, consequently, small‐eyed ‐ primates, such as the tamarins and marmosets, tend to have bright peri‐iridal tissues, while the opposite appears to be the case for the majority of large‐bodied species (Fig. 3; Perea‐García *et al*., 2022). With decreasing size, eyes become less effective for horizontal scanning of the environment. Thus, head orientation, rather than eye movements, are used to facilitate horizontal scanning (Kobayashi & Kohshima, 2001). As a result, the palpebral fissure evolves a more circular shape and peri‐iridal tissues become less exposed to UV radiation in small‐eyed species, lowering the necessity for protective pigmentation. A recent study on a small sample of non‐primate mammals found a negative correlation between eye size and peri‐iridal brightness (Caspar *et al*., 2023; but note that the authors did not distinguish between the nasal and temporal aspect of the eyeball). This tentatively suggests that photoprotective needs may be drivers of ocular pigmentation across mammals, but this finding certainly requires replication with a more inclusive data set.

Interestingly, humans seem to deviate from this pattern, since they are large‐eyed primates that evolved in low‐latitude open habitats but display only weakly pigmented eyes. At the moment, we lack a satisfying explanation for why photoregulatory pressures on humans, or on hominid species in general, which tend to have brighter eyes than closely related catarrhines (Caspar *et al*., 2021; Fig. 3), might be lower than in many other primates. Humans' apparent departure from the general pattern may also prove less pronounced than it currently seems once a comprehensive characterization of phentypic variability in our species is achieved (see Section II.2). In any case, in‐depth comparative work on ocular and orbital morphology and physiology is required to clarify why certain lineages express less protective pigmentation than would be expected. An interesting anatomical trait that such future studies might concentrate on is the position of the limbal crypts at the border between the conjunctiva and the cornea. These structures harbour the limbal stem cells, which constantly replenish the cells of the cornea, enabling proper tissue function. Unlike in domestic pigs (*Sus scrofa*) and laboratory mice (*Mus musculus*), limbal crypts in humans are not evenly distributed in a circular pattern around the iris, but are restricted to its dorsoventral regions and are absent from its lateral aspects (Grieve *et al*., 2015). In living humans, they are almost permanently covered by the eyelids and thus shielded from harmful UV radiation. Therefore, peri‐iridal pigmentation may not be required to effectively protect these structures in our species (Perea‐García *et al*., 2021). Unfortunately, comparative data on non‐human primates are unavailable, so it remains unknown whether the aforementioned morphology of the limbal crypts is a derived trait unique to humans. Apart from mapping limbal crypts, potential differences in the distribution of conjunctival stem cells (i.e. across the bulbar, palpebral, and forniceal region of the conjunctiva) in species with different degrees of pigmentation could be worth exploring. Localizing these cells, however, has proved difficult, even in humans (Ramos *et al*., 2015).

### (2) Sexual selection

The idea of sexual selection as a driver of human ocular appearance has so far almost exclusively been discussed with a focus on iris coloration. The great variance of iris colours in humans (Edwards *et al*., 2016) is indeed an exceptional trait, but certainly not “exclusive” to our species (as claimed by Negro, Carmen Blázquez & Galván, 2017), since distinct eye colours as well as notable continuous variation in iridal coloration have been noted sporadically among other primates (Zhang & Watanabe, 2007; Meyer *et al*., 2013) and in non‐domesticated mammals outside the primate order (Tabin & Chiasson, 2024). To what extent it might be affected by natural rather than sexual selection is a subject of ongoing discussions (Goel, Terman & Terman, 2002; Gründl *et al*., 2012; Suarez, Baumer & Hall, 2021). We highlight here that one can also make a plausible argument for effects of sexual selection on scleral appearance in humans, a subject that has so far remained underexplored (Provine, Cabrera & Nave‐Blodgett, *et al*., 2013; Caspar *et al*., 2021; Wacewicz *et al*., 2022). Indeed, peri‐iridal pigmentation may have been a significant target of sexual selection in our species.

In primates, and possibly various other mammals (e.g. de Oliveira Garcia *et al*., 2021), the brightness of the bulbar conjunctiva decreases over the course of ontogeny. This pattern has so far been quantitatively confirmed in humans [Russell *et al*., 2014; see also qualitative descriptions by Mann (1966) in native Australians], macaques (Perea‐García *et al*., 2024; see also Perrett & Mistlin, 1990), bonobos, and chimpanzees (Perea‐García *et al*., 2019; Clark *et al*., 2023). Thus, bright peri‐iridal tissue complexion can act as an indicator of youth and is evidently perceived as such in our species (Gründl *et al*., 2012; Provine *et al*., 2013; Russell *et al*., 2014). Interestingly, the morphology of the facial portion of the skull in modern humans in general shows strong signatures of paedomorphosis. Hence, various facial traits of adult modern humans are overall more similar to juvenile than to adult fossil hominins and extant great apes (Pérez‐Claros & Palmqvist, 2022). This phenomenon has been suggested to result to some extent from sexual selection, especially on females (Jones, 1995; Puts, 2010), since paedomorphic facial traits are generally more pronounced in women than in men (Jones, 1995; Bulygina, Mitteroecker & Aiello, 2006; Puts, 2010). Across human populations, males strongly favour youthful (or youthful‐looking) female partners (Grammer *et al*., 2003; Puts, 2010), which might relate to the very early onset of menopause in our species (Jones, 1995; Wood *et al*., 2023). Interestingly, available data suggest that this situation is inverse to mate choice patterns in male great apes [chimpanzees (Muller, Thompson & Wrangham, 2006); orangutans (O'Connell, Susanto & Knott, 2020)], which might prioritize more experienced mothers because allomaternal care is negligible in these species when compared to humans (Burkart *et al*., 2009). Experimental studies provide strong evidence that depigmented eyes contribute to a juvenilized facial appearance in humans (Provine *et al*., 2013; Wacewicz *et al*., 2022) and thus complement skull morphology in shaping facial paedomorphosis. Given that humans seem unique among great apes in focusing primarily on the eyes when viewing conspecific faces (Kano *et al*., 2012) and due to the mate‐choice patterns outlined above, we find it likely that ocular phenotypes suggestive of youthfulness may be subject to similar or even greater sexual selection pressure than other facial features. In line with this, preliminary evidence suggests that women tend to have brighter peri‐iridal tissues than men (Kramer & Russell, 2022). Sexual selection is thus a plausible factor that should be considered in discussions on why human ocular pigmentation differs from that of great apes (Caspar *et al*., 2021). Unfortunately, available studies related to this topic have paid no attention to variability in phenotypes and (potentially) perceptual preferences across human populations, posing a limitation on the assessment of sexual selection pressures. It is also unclear to what extent peri‐iridal pigmentation might affect mate choice in other primates. While we consider it plausible that sexual selection was involved in shaping human eye appearance, we lack indications that the same might be the case for species like the pig‐tailed macaque or the golden langur, which convergently evolved bright‐eyed phenotypes (Figs 2 and 3).

### (3) Non‐referential signalling

**Fig. 6:**
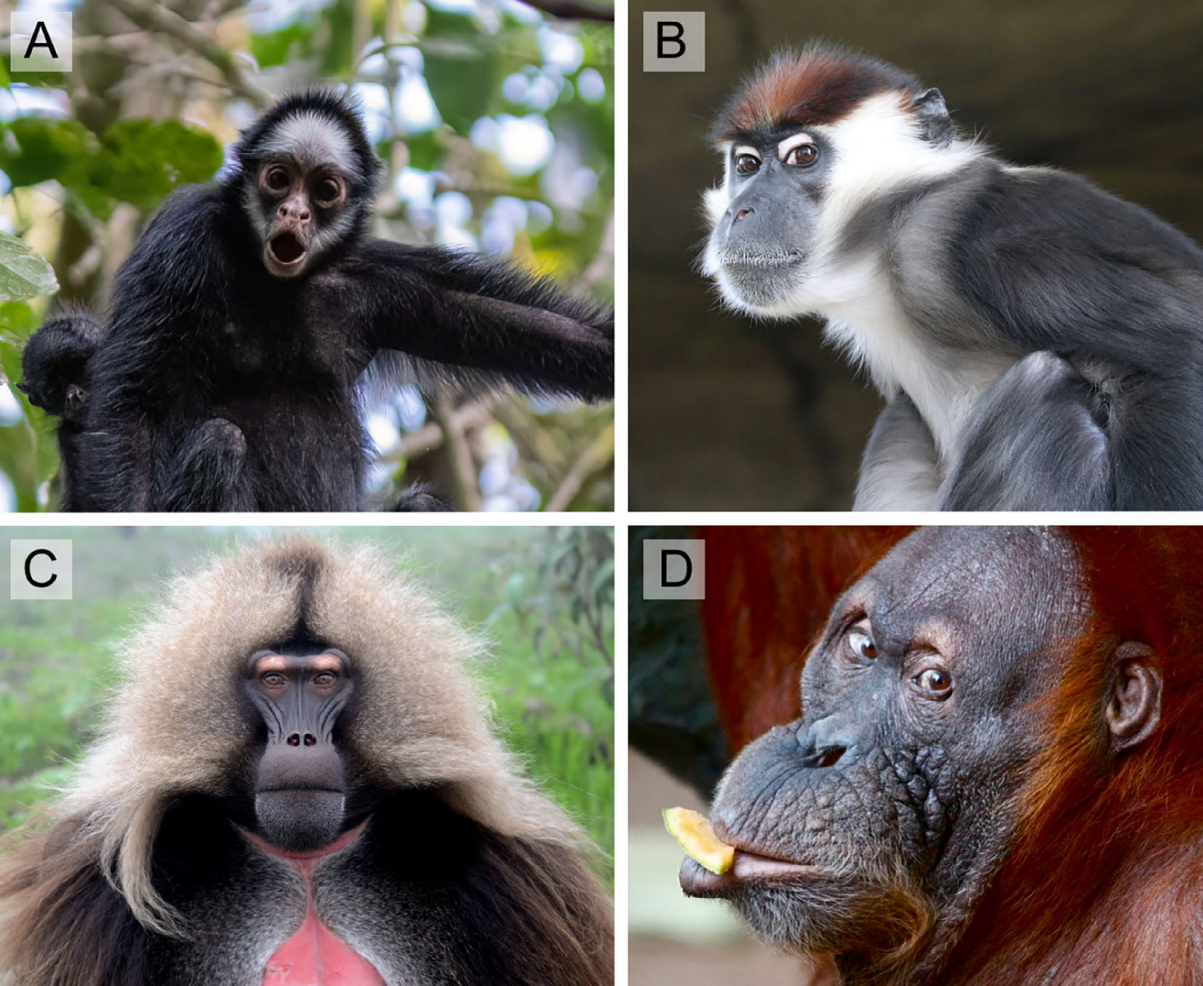
Examples of anthropoid primate species with contrasting facial coloration highlighting the eyes. (A) *Ateles marginatus*, photograph by John Sullivan (iNaturalist, CC BY‐NC, https://creativecommons.org/licenses/by/4.0/). (B) *Cercocebus torquatus*, photograph by Rufus46 (Wikimedia Commons, CC BY‐SA 3.0, https://creativecommons.org/licenses/by‐sa/3.0/deed.de). (C) *Theropithecus gelada*, photograph by Jose Antonio Pascual Trillo (iNaturalist, CC BY‐NC). (D) *Pongo abelii* by William B. Grice (CC BY‐SA 4.0, https://creativecommons.org/licenses/by‐sa/4.0/deed.de). In this species, as in orangutans in general, light eyelids and patches around the eyes are invariably found in juveniles and are sometimes retained into adulthood, as is the case here.

Contrary to traditional notions of “gaze camouflage”, the eyes of many primate species are the most conspicuous region of their face (Kano *et al*., 2022a; see also Whitham *et al*., 2022, 2022) and are often ornamented by contrastingly coloured facial skin or pelage (Fig. 6). This makes them important signalling devices in contexts unrelated to eye‐gaze following. It is obvious that eyes play an important role in shaping various facial expressions, so that the colour of peri‐iridal tissues may well be involved in amplifying a range of ubiquitous social signals [reviewed in Emery, 2000; see Wathan & McComb (2014) for analogous ideas on social communication in ungulates]. Salient ocular pigmentation patterns, as encountered in various primates, could reduce signal ambiguity in agonistic but importantly also affiliative contexts. For instance, mutual eye contact is established to initiate mating in various anthropoid primates [*Alouatta pigra* (Horwich, 1983); *Macaca arctoides* (Linnankoski, Grönroos & Pertovaara, 1993); *Pan paniscus* (Annicchiarico *et al*., 2020)] and, at least in geladas (*Theropithecus gelada*), it has been shown to affect the length of copulation and the frequency of post‐copulatory grooming (Zanoli *et al*., 2021). Unfortunately, empirical data on how peri‐iridal pigmentation might influence the perception of facial expressions in non‐human primates are, to our best knowledge, not available.

It is obvious that facilitating non‐referential communicative signals *via* the eyes is also of importance in our own species. Beyond a relevance to facial gestures, it has been proposed that brightened peri‐iridal tissues in humans may serve as an honest signal of health status, since scleral appearance can be significantly affected by pathologic conditions such as icterus (Roche & Kobos, 2004; Russell *et al*., 2014; Provine *et al*., 2011, 2013). However, we must be cautious to avoid teleological reasoning here: whereas scleral appearance in modern humans is doubtlessly involved in such signalling, we cannot derive a feasible evolutionary pathway for pigment loss from this observation alone. Furthermore, it is unknown if perhaps more subtle changes in the appearance of the eyes can also convey analogous social signals in the presence of more heavily pigmented peri‐iridal tissues.

### (4) Further alternative hypotheses

In addition to the ideas mentioned above, we are aware of three further hypotheses on the evolution of human ocular appearance which we highlight briefly for sake of completeness. These other hypotheses enjoy little to no empirical support (eye appearance as a hybridization barrier) or involve non‐adaptive explanations for phenotypic diversity (genetic drift, de‐pigmentation as a by‐product of selection against aggression). Of these, we consider the genetic drift hypothesis most promising to inform future research.

#### (a) Selection against aggression

It has been proposed that the limited‐to‐absent macroscopic pigmentation in the peri‐iridal tissues of humans and selected other primates (some bonobos, callitrichid monkeys) derives from selection against aggression (“self‐domestication”, reviewed by Sánchez‐Villagra & Van Schaik, 2019), resulting in pleiotropy‐induced pigmentation defects as is typical for the integument of domesticated mammals (Hare, 2017; Mearing *et al*., 2022). While it remains controversial whether humans or other primates have experienced self‐domestication (Sánchez‐Villagra & Van Schaik, 2019; Hecht *et al*., 2023), the available morphological evidence firmly indicates that depigmented peri‐iridal tissues are not a correlate of the domestication process (Caspar *et al*., 2023). Domesticated mammal species can display strongly pigmented eyes (e.g. llamas, yaks, and most horse breeds) and those that show peri‐iridal tissues poor in melanin (e.g. most dog breeds, domestic cats, and rabbits) appear to have inherited this trait from their wild ancestors. This concept thus lacks explanatory power.

#### (b) Species recognition

Another idea is that human scleral appearance originally functioned as an aid for species recognition [Zrzavý *et al*., 2024; hinted at in Emery, 2000; compare Corbett, Brumfield & Faircloth (2024) for an ornithological perspective on eye colour as a species‐recognition device]. Specifically, Zrzavý *et al*. (2024) argued that depigmented peri‐iridal tissues might have emerged to signal species status among sympatric, closely related human species in the geological past, thus preventing hybridization. However, for fossil hominins this idea is essentially non‐falsifiable (even if relevant palaeogenetic data for recently extinct human species such as Neanderthals become available/interpretable at some point) and evidence to suggest that it holds for modern anthropoid primates is lacking. Research on different anthropoid clades indicates that they can rely on more salient pelage characteristics and/or vocalizations to differentiate conspecifics from sympatric heterospecifics (e.g. Santana *et al*., 2012; Allen, Stevens & Higham, 2014). For colour patterns to act as species recognition devices, they would also be expected to show rather constrained intraspecific variation (Bradley & Mundy, 2008). While this is true for peri‐iridal pigmentation within extant human populations, it might not have been the case in fossil hominins and evidently is not in other living species of the hominid family (Mayhew & Gómez, 2015; Perea‐García, 2016; Perea‐García *et al*., 2019; Caspar *et al*., 2021; Clark *et al*., 2023).

#### (c) Genetic drift

Finally, it is worth considering to what degree ocular phenotypes might be shaped simply by genetic drift, in combination with or without an impact of the various selective regimes outlined above (Caspar *et al*., 2021). For the evolution of great apes and humans, drift appears especially relevant, since intraspecific variability of peri‐iridal tissues in this clade appears to be pronounced (Mayhew & Gómez, 2015; Perea‐García, 2016; Perea‐García *et al*., 2019; Caspar *et al*., 2021; Clark *et al*., 2023). Considering the human lineage, we can picture a population of ancestral hominins displaying a diversity of peri‐iridal phenotypes, as in for example modern Western gorillas and bonobos. In these species, scleral appearance covers a spectrum from plain black to almost uniformly white (Mayhew & Gómez, 2015; Perea‐García *et al*., 2019). Simply due to drift phenomena, this ancestral diversity could have been diminished, resulting in the limited spectrum of rather bright‐eyed phenotypes we see in humans today. Compared to great apes, it can be expected that genetic drift played a more pronounced role in the evolution of the human lineage because of rather small effective population sizes in early hominins (e.g. Huff *et al*., 2010), intercontinental migrations (e.g. Manica *et al*., 2007) and severe climate change‐induced bottleneck events (e.g. Schlebusch *et al*., 2020; Muttoni & Kent, 2024, but see Deng, Nielsen & Song, 2024). However such drift effects in humans and other species will remain challenging to test until the genetic underpinnings of primate conjunctival pigmentation have been reasonably characterized.

## IV. CONCLUSIONS

(1) Upon reviewing the evidence supporting the CEH, it is evident that none of its core premises are compellingly substantiated. Nevertheless, research inspired by the CEH has generated several alternative hypotheses concerning the evolution of primate ocular morphology that warrant further exploration. Among these we have highlighted mutually non‐exclusive proposals about photoregulatory functions for peri‐iridal pigmentation, bright scleral appearance as a sexually selected paedomorphic trait, a role of eye pigmentation in non‐referential signalling, and genetic drift. The evolution of human ocular morphology has not been solved; instead we are in an exciting phase of research that needs to consider diverse lines of evidence to make sense of the complex phenotypes at hand.
(2) However, a shortage of phenotypic, behavioural and genetic data limits our ability to test effectively the various ideas discussed herein. At the behavioural level, this comprises the communicative role of eye gaze independent of head orientation in different primate clades, including humans. So far, most human data, including those provided by Tomasello *et al*. (2007), only cover urbanites from Northern Eurasia, which creates numerous well‐known problems (Henrich *et al*., 2010). When considering morphology, peri‐iridal variability in humans from non‐WEIRD populations remains severely understudied and the genetic mechanisms giving rise to the diversity of peri‐iridal pigmentation in humans and other primates remain almost completely obscure.
(3) Addressing these gaps will be crucial to gain a comprehensive understanding of how primate ocular appearance evolved. The proper mapping of peri‐iridal phenotypes and their genetic correlates is also a prerequisite to pave the way for palaeogenetic studies that could provide important insights into the emergence of distinct eye appearances in humans and beyond. Only by appreciating the complex biology and diversity of eye pigmentation across taxa will we be able to improve our understanding of it.
